# Multi‐Harvest Assessment of Physicochemical and Rheological Quality of Wheat Flours From Different Processors in Southern Brazil

**DOI:** 10.1111/1750-3841.71033

**Published:** 2026-03-29

**Authors:** Andressa Centenaro, Eunice Valduga, Clarice Steffens

**Affiliations:** ^1^ Department of Food Engineering URI ‐ Erechim Erechim Brazil

**Keywords:** dough rheology, grain industry, physicochemical properties, *Triticum aestivum* L., wheat processing quality

## Abstract

This study evaluated the physicochemical and rheological characteristics of wheat flours sourced from different processors in southern Brazil, covering the harvest seasons from 2021 to 2025. Rheological analysis is essential for predicting dough behavior during processing and ensuring the quality of flours. Samples were evaluated in relation to alveographic tests that measured gluten strength (*W*), tenacity (*P*), and extensibility (*L*), as well as gluten content, Falling Number (*FN*), moisture, mineral content (ash), and color parameters (L*, a*, and b*). High values for *W*, *P*, *L*, gluten content, and *FN* showed strong flours with good enzymatic stability and a well‐developed protein structure. These characteristics are not desirable for biscuit doughs, which require lower resistance and greater extensibility. These findings reflected the edaphoclimatic conditions and the predominant cultivars in the region. The comparative analysis among processors and harvest seasons showed variability in quality parameters. Some processors demonstrated positive progress, producing lighter and stronger flours with improved rheological performance in the 2024 and 2025 seasons. Other processors showed greater variability within the same harvest, indicating the need for adjustments in industrial processing. Multivariate analysis (PCA) identified flour strength, gluten index, and *FN* as the most important variables for distinguishing processors and harvest seasons. The results support the strategic selection of suppliers and the development of specific formulations, contributing to improved control and standardization across the industry.

**Practical Applications:**

Physicochemical and rheological variability of wheat flours supplied by different processors across harvest seasons directly affects dough performance and industrial process stability. The identification of flour strength, gluten characteristics, and enzymatic activity as critical quality parameters supports more accurate supplier qualification and targeted formulation adjustments. The multivariate approach enables efficient monitoring of quality fluctuations associated with harvest year and climatic conditions, contributing to improved process control and risk management in biscuit manufacturing.

## 1 Introduction

The technological characterization of wheat (*Triticum aestivum* L.), based on its structural, processing, and chemical composition attributes, is essential for determining its industrial applicability. Among the most relevant parameters, rheological analyses are notable, since they allow the evaluation of dough behavior under controlled conditions prior to its actual use in production processes and specific formulations (Bueno et al. 2016; Scheuer et al. 2011; Ziegler et al. 2025). These analyses provide critical information for predicting the technological performance of flours and, consequently, the quality of derived products.

However, although wheat flour quality has been widely studied, the introduction did not clearly define the central technological problem faced by the milling and processing industry in Southern Brazil. In this region, climatic instability during grain filling and preharvest periods frequently leads to significant variability in enzymatic activity and gluten performance, directly affecting industrial suitability. This variability represents a critical bottleneck for processors who require consistent raw material quality for standardized biscuit and pasta production.

Because starch accounts for approximately 70%–75% of the wheat kernel and is the main determinant of hydration, viscosity development, and dough viscoelasticity, understanding starch functionality is fundamental in assessing flour technological behavior (Mustafa et al. 2025). Starch properties (granule integrity, swelling capacity, susceptibility to α‐amylase) are particularly relevant for predicting processing quality, especially under conditions where environmental variability may affect grain physiology.

The rheological properties of wheat flour are directly related to dough viscosity, elasticity, and water absorption capacity, which influence the volume, texture, and appearance of final products. Tests such as the Chopin alveograph, gluten quantification, Falling Number (*FN*), and color analysis are widely applied to measure characteristics that include tenacity (*P*), extensibility (*L*), flour strength (*W*), and enzymatic activity (Nkurikiye et al. 2024; Wang et al. 2025). These indicators assist in determining the suitability of a flour for specific applications such as breadmaking, confectionery, or biscuit production, each of which requires distinct technological properties. Flour quality analysis through fundamental rheological methods in nonlinear viscoelastic regions provides more accurate predictors of industrial performance than traditional empirical assays (Yazar 2023). Additionally, the suitability of flours for breadmaking has been shown to depend strongly on gluten quality and quantity, as well as on the ability of the protein network to maintain cohesion under severe deformation.

The FN method is commonly used as an indirect indicator of α‐amylase activity, reflecting starch degradation through viscosity reduction during gelatinization. Low *FN* values are generally associated with preharvest sprouting and excessive α‐amylase activity, which can compromise dough structure, product texture, and processing stability. On the other hand, excessively high *FN* values may indicate insufficient enzymatic activity, which can also negatively affect certain applications. Therefore, maintaining *FN* within an optimal range is essential for industrial performance (Ben Mariem et al. 2021).

Although less explored than breadmaking, the biscuit industry has also shown growing evidence that specific flour characteristics are determinants of processing efficiency. For example, a study on biscuit dough partially substituted with spelt flour found that dough viscosity decreased as the proportion of spelt increased, which favored expansion and improved surface characteristics in finished biscuits (Mykhailo et al. 2021). Another study evaluating the incorporation of wheat bran and arabinoxylans in molded biscuits showed that arabinoxylans significantly affected the elastic response of the dough and influenced starch gelatinization during baking (Molina et al. 2021). Mixing time alters the macrostructure of the protein network and is strongly correlated with biscuit texture, hardness, and fragility (Dhal et al. 2023).

For wheat flours intended for biscuit manufacturing, it is well established that more extensible doughs with lower tenacity, moderate gluten content, and reduced flour strength tend to promote greater spread and more desirable textural properties. A recent comparison between ancient and modern wheat types showed that cultivars with lower water absorption and retention capacity produced biscuits with larger diameters, softer textures, and greater expansion during baking (Methe et al. 2024).

For biscuits, weaker gluten networks are generally preferred, as strong gluten development may limit spread and result in harder textures. In contrast, pasta processing requires sufficient protein content and gluten strength to ensure structural integrity during extrusion and cooking. Therefore, the technological interpretation of flour quality parameters must always consider the intended end use (Li et al. 2026). A property that is undesirable for one product category may be acceptable or even beneficial for another.

Several strategies have been proposed in previous research to mitigate quality variability. At the agricultural level, breeding programs have focused on developing cultivars with improved tolerance to preharvest sprouting and greater technological stability under environmental stress. At the postharvest stage, grain segregation and classification systems are used to separate lots according to quality standards. Within mills, flour blending is commonly employed to standardize *FN* values and protein content. Additionally, controlled supplementation with fungal α‐amylase can correct excessively high *FN* values, while oxidizing or reducing agents are used to adjust gluten strength. In the biscuit industry, dilution of strong flours with corn or cassava starch is frequently applied to reduce gluten strength and optimize spread characteristics. Although these corrective approaches are widely used, they represent adjustments to variability rather than definitive solutions.

In southern Brazil, the country's main wheat‐producing region, monitoring the technological quality of flours is essential for ensuring the competitiveness of the wheat value chain and meeting the demands of the food industry (Conab 2024). Studies have shown that variations in the quality of flours produced in different regions are associated with genetic factors, edaphoclimatic conditions, and technological aspects such as cultivar type, processor characteristics, and grain storage practices (Dahmer et al. 2018; Junges et al. 2025; Reznick et al. 2021). Although most flours exhibit characteristics suitable for breadmaking, many do not present ideal properties for biscuit production, which requires more extensible and less resistant doughs, with lower gluten content and reduced flour strength.

Recent climatic fluctuations in southern Brazil, marked by excessive rainfall during grain maturation, temperature anomalies, and altered humidity regimes, have directly affected starch quality (SECOM 2024). These conditions are known to promote kernel sprouting, increase α‐amylase expression, reduce starch integrity, and intensify year‐to‐year variability in flour functional properties (Han et al. 2025; Shi et al. 2024). Understanding how such climate‐driven changes propagate through technological indicators such as *FN*, *W*, *P/L*, extensibility, and gluten behavior is fundamental for the cereal industry.

Given this scenario, systematic evaluation of the physicochemical and rheological behavior of wheat flours processed in southern Brazil across multiple harvest seasons is necessary. This approach allows the identification of quality patterns, interannual variations, and potential industrial adjustments, including the use of enzymes and technological aids that may improve flour performance for specific products (Ferreira et al. 2025).

Furthermore, there is still limited integrated information evaluating how key technological parameters, such as *FN*, moisture, ash content, and gluten strength, vary among processors and harvest years within this regional context, and how these variations impact biscuit and pasta applications. A clearer understanding of these relationships is essential for improving raw material selection, optimizing industrial performance, and supporting decision‐making across the wheat production chain.

Although previous studies have investigated wheat quality in Brazil, most have focused on isolated harvests, specific cultivars, or single technological parameters, predominantly in the context of breadmaking. Integrated, multi‐harvest evaluations that simultaneously consider physicochemical attributes, rheological behavior, processor‐level variability, and their implications for biscuit production remain scarce, particularly in southern Brazil, where climatic instability intensifies year‐to‐year functional variability.

In this context, a systematic and comparative assessment across consecutive harvest seasons becomes essential to determine whether flours marketed for biscuit production actually meet the technological requirements of this sector and to identify the main factors driving variability among processors.

Therefore, this study aimed to evaluate and compare the physicochemical and rheological profiles of wheat flours produced by different processors in southern Brazil over five consecutive harvest seasons (2021–2025), integrating conventional quality parameters, alveographic measurements, and multivariate analysis to identify the principal variables responsible for inter‐harvest and inter‐processor variability and to assess their suitability for biscuit manufacturing.

## 2 Materials and Methods

### 2.1 Sample Collection and Preparation

**FIGURE 1:**
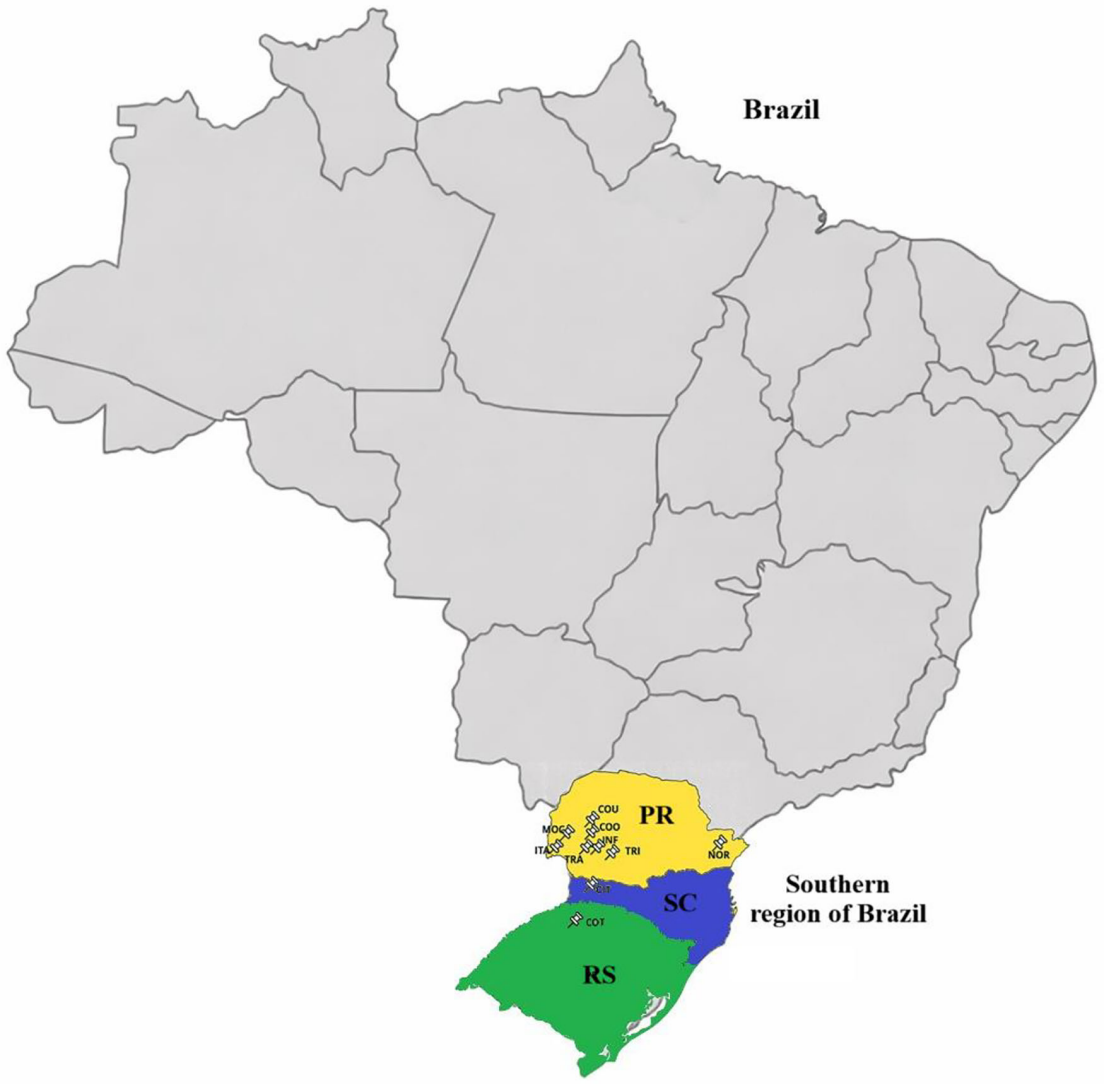
Geographical location of the wheat flour processors selected in southern Brazil.

This study was conducted in industrial unit, using 20–50 independent common wheat flour samples (*T. aestivum* L.), depending on the supplier and harvest season, collected directly from the raw material reception stage of a commercial biscuit and pasta manufacturing plant located in western Paraná, Brazil. The evaluated flours were supplied by 10 industrial wheat processors located in the southern region of Brazil (Figure 1; Table 1), covering the 2021/2022 to 2024/2025 harvest seasons.

**TABLE 1:** Wheat flour processors in southern Brazil and analyzed harvests.

Processors	Code	Region	Harvests
1	COO	West‐PR	2021/2022, 2022/2023
2	MOC	West‐PR	2021/2022, 2022/2023
3	COT	Northwest‐RS	2022/2023, 2023/2024
4	TRA	West‐PR	2023/2024
5	INF	West‐PR	2021/2022, 2023/2024
6	CIT	West‐SC	2022/2023, 2023/2024
7	NOR	East‐PR	2022/2023, 2023/2024, 2024/2025
8	ITA	West‐PR	2021/2022, 2022/2023, 2023/2024, 2024/2025
9	COU	West‐PR	2023/2024
10	TRI	Central‐West‐PR	2021/2022, 2022/2023, 2023/2024, 2024/2025

The selection of processors considered geographic diversity, industrial relevance, and representativeness of the harvests, aiming to capture interannual and regional variability in wheat flour quality associated with edaphoclimatic conditions and industrial milling practices. The processors were located in the western region of Paraná (PR), central‐west Paraná, northwest of Rio Grande do Sul (RS), and western Santa Catarina (SC), as detailed in Table 1.

Wheat flour samples intended for biscuit production were randomly collected from 1000‐kg bulk bags (big‐bags) during the industrial reception and quality control stage, prior to processing. Sampling was performed at the biscuit and pasta industry according to the number of big‐bags received from each processor, following the sampling plan and inspection procedures for attributes established by guidelines for cereal grains (ICC Standard No. 101/2 1995 [ICC 1995]). A grain probe (Model 5301–9, J. Prolab, Brazil) was inserted at different points in each big‐bag to ensure a representative sampling of the entire lot.

After collection, flour portions were manually homogenized to ensure uniformity and representativeness, minimizing variability prior to laboratory analyses. The quartering technique was applied to obtain analytical aliquots, while the remaining material was retained as a backup sample for potential reanalysis or verification. The samples were packed in hermetically sealed plastic bags and stored in a dry and cool environment until analysis, in order to prevent contamination or physicochemical alterations.

Samples obtained from different industrial lots (with lot sizes ranging from 20 to 50 samples, depending on the supplier and harvest season) were characterized in terms of physicochemical properties (moisture, ash content, and color parameters L*, a*, and b*) and rheological properties, including enzymatic activity by *FN*, alveographic parameters (*W*, *P*, and *L*), and gluten content.

### 2.2 Physicochemical Characterization

Moisture was determined according to the methodology of the Instituto Adolfo Lutz (IAL 2008). Approximately 3.0 g of sample was weighed in a porcelain crucible and dried in an air recirculating oven (Fanem, model 320‐SE, Brazil) at 105°C for approximately 4 h and/or until constant weight was obtained. After drying, the samples were cooled in a desiccator for 30 min and reweighed. The drying and weighing procedure was repeated until a constant weight was achieved.

Color measurements were performed using a portable colorimeter (CR‐400, Konica Minolta, Osaka, Japan). The instrument was calibrated prior to each set of measurements using the standard white calibration plate supplied by the manufacturer. Measurements were carried out under illuminant D65, with a 2° standard observer, an 8 mm measurement aperture, and a 0° viewing angle geometry. Light was emitted onto the sample surface, and the reflected light was recorded to determine the color parameters: L* (lightness, 0 = black, 100 = white), a* (green–red axis), and b* (blue–yellow axis).

The total mineral content (ash) was determined following the methodology of the Instituto Adolfo Lutz (IAL 2008). Samples (approximately 3.0 g weighed in porcelain crucibles) were pre‐carbonized over a low Bunsen flame until cessation of smoke formation and then incinerated in a muffle furnace (SP Labor, model SP‐1200, Brazil) at 550°C for approximately 6 h, until complete oxidation of organic matter was achieved and a light gray ash was obtained. After incineration, the crucibles were placed in a desiccator, cooled to room temperature, and weighed.

### 2.3 Rheological Analyses

The gluten content was determined using a Glutomatic system (Perten Instruments, Sweden). After verifying the cleaning and lubrication of the equipment, the washing chamber was assembled with a moistened polyester sieve (88 µm pore size). A 10‐g sample was mixed with 4.8 mL of saline solution (2%) and automatically washed for 300 s after a 20‐s premixing step, using approximately 265 mL of the saline solution. The gluten formed was weighed, and part of it was centrifuged (Fanem, model 206‐B, Brazil) at 3000 × *g* for 5 min. The sum of the retained and collected gluten fractions represented the wet gluten. Subsequently, the gluten was dried in a Glutork (Perten Instruments, Model 2020 with Glutimer GT2020) at 150°C for 4 min, cooled in a desiccator to room temperature, and weighed to obtain the dry gluten.

For the FN determination, the sample was weighed according to its moisture content. Then, 25 mL of distilled water (22°C ± 2°C) was added to a viscometric tube, and the sample was transferred using a funnel to avoid spillage. The tube was sealed and shaken vigorously (20–30 times) to form a homogeneous suspension. The viscometric rod was used to reincorporate any material adhering to the tube walls. Within 30 s, both the tube and the rod were placed into the water bath of the Falling Number apparatus (Falling Number 1700, Perten Instruments, Hägersten, Sweden). After 5 s of rest, automatic stirring was initiated for 60 s to ensure complete gelatinization, followed by the release of the rod in free fall. The time required for the rod to reach the bottom was automatically recorded as the *FN* value, which indirectly reflects α‐amylase activity in the sample.

The alveographic parameters were determined using an Alveograph NG (Chopin Technologies, Villeneuve‐la‐Garenne, France), equipped with the Alveolink software. Approximately 250 ± 0.5 g of flour was mixed with a saline solution (0.5% NaCl, w/v) for 8 min at room temperature (22°C ± 2°C). The dough was divided into five standardized pieces, laminated, and molded into disks using a calibrated mold of 40 mm diameter in a defined sequence. The disks were conditioned on resting plates and kept for 28 min in a temperature‐controlled chamber at 25°C with 60% relative humidity. Each disk was then inflated with air until rupture, generating a curve from which *P* (tenacity, mmH_2_O), *L* (extensibility, mm), and *W* (dough strength, × 10^−4^ J) were calculated.

### 2.4 Statistical Analysis

A total of 20–50 flour samples (depending on the supplier and harvest season) were included in the statistical analysis. The results obtained from the samples were analyzed using one‐way analysis of variance (ANOVA), considering the different harvests. The means of the results were compared using Tukey's test at a 95% confidence level. Additionally, principal component analysis (PCA) was performed to investigate the structure and correlation between the physicochemical and rheological variables of the samples, with the aim of reducing data dimensionality and identifying patterns or clusters in the properties of the wheat flour samples. All variables included in the PCA were previously standardized to avoid scale effects and to ensure comparability among parameters with different measurement units. The analyses were carried out using Past version 4.03 software.

## 3 Results and Discussion

### 3.1 Comparative Analysis of the Physicochemical and Rheological Characteristics of Wheat Flours From Southern Brazil Across the 2021/2022 to 2024/2025 Harvests

**FIGURE 2:**
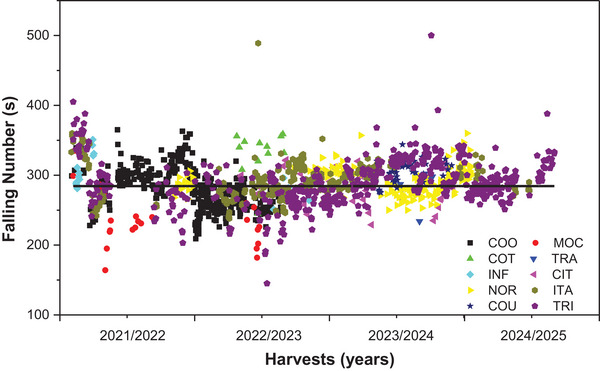
Falling Number (*FN*) values of wheat flours from the 2021/2022 to 2024/2025 harvests, produced by different processors (COO, COT, INF, NOR, COU, MOC, TRA, CIT, ITA, and TRI) in the southern region of Brazil.

Figure 2 shows the *FN* values of wheat flours obtained from different processors in Southern Brazil between the 2021/2022 and 2024/2025 harvests. In most cases, *FN* values ranged from 250 to 350 s, an interval considered adequate for breadmaking flours, according to international technical standards (AACC 2000). This range is likely a consequence of industrial selection practices: when purchasing wheat, mills establish minimum *FN* thresholds and reject lots with lower values, which explains why extremely low *FN* measurements are absent from the dataset.

Values below 250 s indicate high α‐amylase activity, often associated with premature grain germination, which may lead to reduced baking quality, lower water absorption, and decreased loaf yield. Conversely, values above 350 s suggest low enzymatic activity, which can impair fermentation and result in dense, poorly developed crumb structures (Delcour and Hoseney 2010).

The results were consistent with those reported in the literature, with *FN* values between 281 and 306 s in flours intended for bread and biscuit production, confirming the technological suitability of most of the analyzed samples (Costa et al. 2008).

Regarding processor performance, the COO exhibited stable *FN* values over the 5‐year period, with an average around 300 s, reflecting consistency in both raw material quality and processing conditions. In contrast, the COU showed greater variability from 2023/2024 onward, with samples falling outside the ideal range, possibly due to differences in crop origin or seasonal variations. The COT, INF, and NOR presented intermediate behavior, with values centered around the mean but occasional deviations, likely influenced by seasonal factors such as rainfall patterns during harvest (Conab 2024; Costa et al. 2008).

The TRA, TRI, and MOC displayed the lowest *FN* values, particularly between 2022/2023 and early 2023/2024. This pattern may be related to adverse weather conditions, including increased preharvest rainfall, which promotes grain sprouting in the field and enhances α‐amylase activity (INMET 2022).

From an industrial perspective, although most *FN* values fall within ranges acceptable for breadmaking, they are not necessarily optimal for biscuit production, where excessive enzymatic stability may limit starch modification during baking. Therefore, interpreting *FN* values solely based on breadmaking standards may overlook application‐specific requirements.

Overall, the longitudinal analysis highlights not only inter‐harvest variability but also the influence of management practices, storage conditions, and cultivar selection on the rheological properties of wheat flours. Such variation underscores the importance of continuous monitoring of technological quality parameters, such as the *FN*, in the industrial quality control of flours intended for different end uses.

**FIGURE 3:**
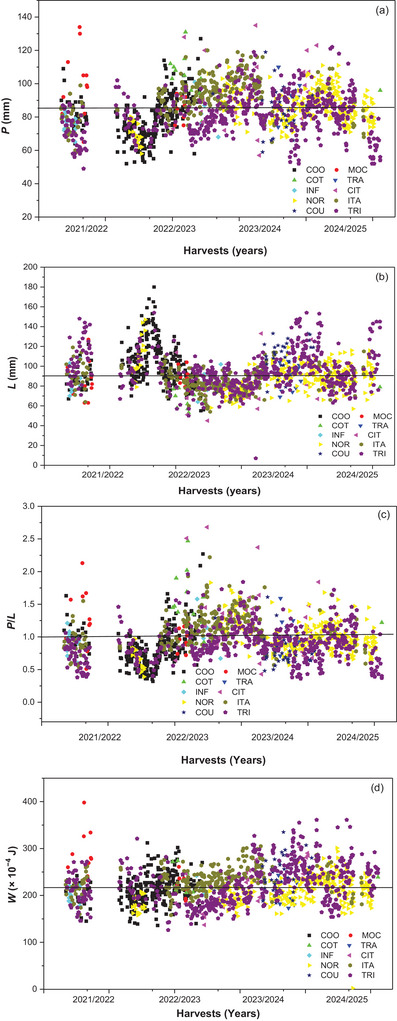
Alveograph results showing dough tenacity (*P*) (a), extensibility (*L*) (b), tenacity to extensibility ratio (*P/L*) (c), and gluten strength (*W* [× 10^−4^ J]) (d) of wheat flours from the 2021/2022 to 2024/2025 harvests, produced by different processors (COO, COT, INF, NOR, COU, MOC, TRA, CIT, ITA, and TRI) in the southern region of Brazil.

Figure 3 presents the alveograph results of wheat flours collected between the 2021/2022 and 2024/2025 harvests, considering *P*, *L*, the *P/L* ratio, and overall baking strength *W*. These parameters describe dough behavior during fermentation and gluten structure development and are widely used to assess the technological suitability of flours (Chen et al. 2024). Values of *W* ranged between 160 × 10^−4^ J and 340 × 10^−4^ J, characterizing medium‐ to high‐strength flours suitable for breadmaking, laminated doughs, and fermented biscuits. Flours with *W* above 300 × 10^−4^ J indicate the presence of high‐quality proteins and a more elastic gluten network, whereas those below 200 × 10^−4^ J are considered weaker and more appropriate for products with short fermentation times (Marti et al. 2013).

Among the evaluated processors, ITA, COO, and NOR showed mean *W* values above 300 × 10^−4^ J, demonstrating technological stability. However, for biscuit manufacturing, excessively high *W* values may result in doughs with reduced spreadability and increased hardness after baking. Thus, the predominance of medium‐ to high‐strength flours suggests a technological profile more aligned with breadmaking than with biscuit production.

The TRA, though with lower mean strength, displayed low variability among harvests, suggesting uniform raw material sourcing. Conversely, the COU and MOC exhibited a downward trend in *W* over the last harvests, possibly linked to reduced protein quality of the wheat cultivars or unfavorable environmental conditions during grain filling (Filip et al. 2023).

Analysis of *P* and *L* showed complementary behavior: flours with high *P* values (>100 mm) and low *L* values (<80 mm) yielded stronger, less extensible doughs. Such characteristics are advantageous for bread and certain pasta products but may impair conformability in molded or laminated biscuits. In contrast, processors with higher *L* and lower *P*/*L* ratios produced more balanced flours, ideal for biscuits and industrial doughs.

The *P/L* ratio proved to be an effective indicator of the balance between dough strength and elasticity. Ratios close to 1.0 represent ideal proportions for fermented bread doughs, whereas values above 1.5 indicate excessively tenacious and less extensible doughs. ITA and NOR maintained *P/L* ratios between 0.8 and 1.2, while COU and COT occasionally exceeded 1.5 in certain harvests.

These results reinforce that rheological suitability depends on the target product and that strength alone does not define flour quality. Instead, the balance between tenacity and extensibility is critical for predicting industrial performance.

**FIGURE 4:**
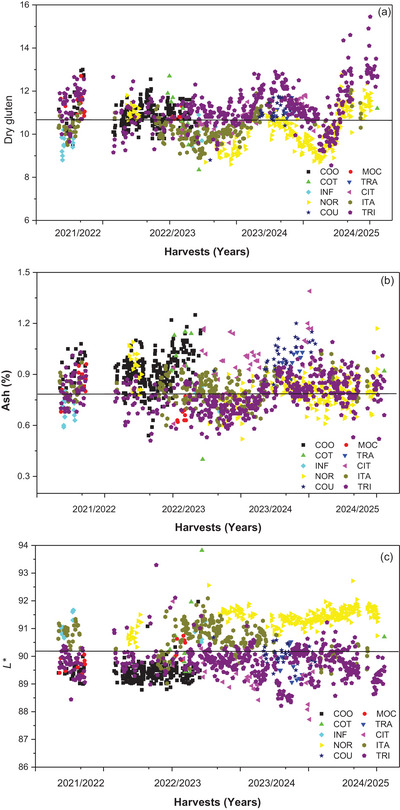
Comparative results of dry gluten (a), ash content (b), and L* color (c) of wheat flours from the 2021/2022 to 2024/2025 harvests, produced by different processors (COO, COT, INF, NOR, COU, MOC, TRA, CIT, ITA, and TRI) in the southern region of Brazil.

The analysis of gluten content (wet and dry) over recent years in southern Brazil reveals a predominance of values ranging from 9% to 14%, with a gradual increasing trend across harvests (Figure 4). This behavior reflects the characteristics of wheat cultivars traditionally grown in the region, which are selected primarily to meet breadmaking requirements, demanding higher protein and gluten levels to ensure dough structure and gas retention (Costa et al. 2017; Scheuer et al. 2011).

When compared to the parameters recommended for flours intended for biscuit production, these values are considerably above the ideal range. Flours used for biscuit manufacturing should have low gluten contents, preferably between 7% and 9%, to promote a weak gluten network (Brazil 2005). Such characteristics result in doughs that are more extensible and easy to shape, with low elasticity and minimal unwanted expansion during baking.

The high dry gluten content identified in wheats from southern Brazil compromises desirable attributes for the biscuit industry, such as greater friability, light texture, and reduced hardness. Excess gluten promotes excessive development of the protein network, leading to tougher and more elastic doughs, undesirable features for laminated, molded, or extruded biscuits, which require doughs with lower cohesion and resistance (Dizlek et al. 2022).

Overall, the analyzed data indicate that, in most cases, wheats produced in southern Brazil do not meet the optimal requirements for biscuit manufacturing. Nevertheless, Brazilian biscuit manufacturers successfully manage gluten strength by employing specific strategies, such as the addition of dough conditioners and emulsifiers, or by diluting wheat flour with alternative starches, such as corn or cassava, to achieve the desired dough extensibility and handling properties. Therefore, the apparent mismatch between flour profile and biscuit requirements does not necessarily prevent industrial application but requires technological correction during formulation.

The analysis of ash content shows that most values are concentrated between 0.55% and 0.90%, with only slight variations across harvest years (Figure 4b). This behavior is consistent with the characteristics of wheat cultivated in the region, which is typically milled for general‐purpose or bread flours. These products often present slightly higher ash contents due to the extraction of a greater proportion of outer grain layers during milling (Evlice et al. 2024).

When compared with the ideal parameters for flours intended for biscuit production, the observed values are generally slightly above the recommended range. Suitable flours for biscuit manufacturing should exhibit lower ash contents, typically below 0.55%, criteria established by the Brazilian Technical Instruction No. 8 (Brazil 2005). This parameter reflects flour purity and a lower proportion of bran particles, resulting in better color, softer texture, and improved sensory quality in the final product. However, modern consumers are increasingly prioritizing health‐conscious products, which has led the biscuit industry to incorporate flours with higher ash content, such as whole wheat or partially refined flours. Since ash content is a proxy for bran, fiber, and other bioactive components of the wheat kernel, using such flours can enhance the nutritional value of biscuits, despite slightly compromising traditional technological properties such as color and texture. This trend reflects a trade‐off between product quality and nutritional enrichment, which is becoming more relevant in current consumer markets.

Flours with higher ash contents tend to have a darker color and may negatively affect biscuit texture, leading to products that are firmer or less delicate in appearance, undesirable attributes for most biscuit quality standards (Costa et al. 2008). Consequently, the data indicate that a large portion of the analyzed samples do not fully meet the ideal specifications for biscuit flours, being more suitable for breadmaking or general‐purpose applications.

The color analysis, particularly the L* parameter, not only reflects the whiteness of the flour but is also influenced by soil characteristics and grain quality. As shown in Figure 4c, the NOR exhibited higher L* values compared to the TRI, COU, and TRA from 2023/2024 to 2024/2025, with values above 91. In contrast, the COO showed L* values between 89 and 90 in the second half of 2022/2023, reinforcing that climatic conditions, particularly heavy rainfall recorded in southern Brazil, can significantly affect wheat quality (INMET 2022).

### 3.2 Comparative Analysis of the Physicochemical and Rheological Characteristics of Wheat Flours From Different Regions Across the 2021/2022 to 2024/2025 Harvests

Tables 2 and 3 present the physicochemical characteristics (total minerals—ash content and color: L*, a*, b*) and rheological parameters (alveograph: *W* [× 10^−4^ J], *P*, and *L*; *FN*; and gluten properties) of wheat flours intended for biscuit production from the southern region of Brazil, across the 2021/2022 to 2024/2025 harvests and from different processors.

**TABLE 2:** Physicochemical and rheological characteristics of wheat flours from different processors (COO, MOC, INF, ITA, and TRI) from the 2021/2022 to 2024/2025 harvests.

Characteristics		Processors of wheat flours/harvests													
		COO		MOC		INF		ITA				TRI			
		2021/2022	2022/2023	2021/2022	2022/2023	2021/2022	2023/2024	2021/2022	2022/2023	2023/2024	2024/2025	2021/2022	2022/2023	2023/2024	2024/2025
Alveograph	*P* (mm)	78.9^a^ (10.3)	77.6^a^ (13.2)	100.4^a^ (9.5)	84.6^b^ (7.3)	77.3^b^ (5.5)	82.8^a^ (6.4)	80.0^b^ (9.3)	95.2^a^ (9.4)	95.7^a^ (11.6)	77.0^b^ (7.0)	73.7^b^ (5.7)	81.9^ab^ (4.5)	86.3^a^ (6.7)	78.3^b^ (6.9)
	*L* (mm)	94.9^b^ (15.7)	107.6^a^ (23.9)	86.5^a^ (9.1)	88.6^a^ (9.7)	85^a^ (10.8)	83.6^a^ (14.6)	93.6^a^ (8.0)	81.5^b^ (8.1)	90.8^a^ (9.4)	91.7^a^ (9.6)	105.5^a^ (13.4)	84.4^c^ (12.5)	100.1^ab^ (10.3)	94.2^b^ (12.0)
	*P*/*L*	0.87^a^ (0.23)	0.78^a^ (0.30)	1.2^a^ (0.47)	0.97^a^ (0.18)	0.93^a^ (0.16)	1.0^a^ (0.35)	0.88^b^ (0.22)	1.2^a^ (0.25)	1.1^a^ (0.28)	0.86^b^ (0.20)	0.73^b^ (0.22)	1.0^a^ (0.27)	0.90^ab^ (0.26)	0.88^ab^ (0.27)
	*W* (× 10^−4^ J)	202.8^b^ (26.5)	216.5^a^ (32.2)	283.7^a^ (52.3)	219.2^b^ (22.8)	200.0^a^ (15.3)	208.8^a^ (21.2)	218.1^bc^ (25.4)	231.0^b^ (24.7)	256.1^a^ (25.3)	201.9^c^ (20.9)	215.1^b^ (24.7)	189.4^b^ (27.1)	246.5^a^ (23.5)	239.9^a^ (18.1)
	*Ei* (%)	44.4^a^ (4.4)	45.4^a^ (4.2)	55.8^a^ (5.8)	46.6^b^ (4.6)	48.4^a^ (2.8)	46.4^a^ (1.7)	48.5^a^ (2.2)	45.6^b^ (2.8)	48.6^a^ (2.0)	45.8^b^ (1.8)	53.6^a^ (17.9)	41.0^b^ (4.0)	52.1^a^ (14.9)	56.2^a^ (8.9)
FN	*FN* (s)	289.4^a^ (31.6)	285.6^a^ (32.4)	260.3^a^ (29.1)	225.0^b^ (28.7)	310.8^a^ (24.2)	271.8^b^ (17.4)	299.6^ab^ (18.6)	286.8^b^ (15.1)	307.8^a^ (10.7)	279.4^c^ (5.8)	295.8^ab^ (22.4)	266.8^c^ (27.9)	306.6^a^ (11.7)	293.6^b^ (11.9)
Gluten	Wet (%)	31.8^a^ (1.7)	32.1^a^ (1.3)	32.7^a^ (1.1)	32.4^a^ (0.87)	27.8^b^ (1.2)	29.9^a^ (1.6)	29.9^b^ (1.4)	30.0^b^ (1.4)	31.8^a^ (1.4)	33.2^a^ (1.6)	32.3^b^ (2.5)	34.5^a^ (2.1)	33.6^ab^ (2.4)	34.5^a^ (2.4)
	Dry (%)	10.9^a^ (0.79)	10.9^a^ (0.49)	11.4^a^ (0.55)	10.7^b^ (0.27)	9.5^b^ (0.38)	10.1^a^ (0.58)	10.3^bc^ (0.46)	9.9^c^ (0.42)	10.7^ab^ (0.45)	11.3^a^ (0.65)	11.1^bc^ (0.87)	10.9^c^ (0.52)	11.2^b^ (0.80)	11.9^a^ (1.4)
	Index (%)	98.3^a^ (0.70)	97.6^a^ (1.0)	97.0^a^ (1.5)	93.8^b^ (3.9)	94.8^b^ (1.6)	97.7^a^ (0.85)	97.9^a^ (1.0)	97.6^ab^ (1.4)	96.5^bc^ (1.2)	96.3^c^ (1.1)	97.9^a^ (1.5)	87.9^c^ (7.7)	93.8^b^ (5.4)	96.0^a^ (1.9)
Ash	Wet (%)	0.80^a^ (0.07)	0.78^a^ (0.10)	0.74^a^ (0.07)	0.58^b^ (0.04)	0.60^b^ (0.05)	0.67^a^ (0.06)	0.71^a^ (0.05)	0.69^a^ (0.06)	0.73^a^ (0.09)	0.75^a^ (0.06)	0.69^b^ (0.08)	0.62^c^ (0.06)	0.72^a^ (0.08)	0.72^a^ (0.08)
	Dry (%)	0.92^a^ (0.08)	0.90^a^ (0.12)	0.85^a^ (0.08)	0.67^b^ (0.05)	0.68^b^ (0.06)	0.78^a^ (0.07)	0.81^a^ (0.06)	0.80^a^ (0.07)	0.84^a^ (0.10)	0.85^a^ (0.08)	0.79^b^ (0.01)	0.71^c^ (0.07)	0.83^ab^ (0.08)	0.82^a^ (0.09)
Color	L*	89.5^a^ (0.25)	89.4^a^ (0.34)	89.7^a^ (0.22)	90.4^a^ (0.37)	91.1^a^ (0.39)	90.8^a^ (0.29)	90.8^a^ (0.33)	90.7^a^ (0.53)	90.5^ab^ (0.45)	90.1^b^ (0.34)	89.8^ab^ (0.61)	89.9^a^ (0.44)	89.7^bc^ (0.62)	89.6^c^ (0.46)
	a*	0.32^a^ (0.13)	0.36^a^ (0.16)	0.27^a^ (0.15)	0.01^b^ (0.001)	−0.07^a^ (0.10)	0.03^a^ (0.12)	−0.02^b^ (0.15)	0.02^b^ (0.15)	0.11^a^ (0.20)	0.04^b^ (0.13)	0.10^b^ (0.19)	−0.01^c^ (0.13)	0.20^a^ (0.21)	0.21^a^ (0.15)
	b*	11.8^a^ (0.24)	11.6^a^ (0.39)	11.6^a^ (0.24)	11.2^a^ (0.29)	11.5^a^ (0.20)	11.0^b^ (0.40)	11.4^ab^ (0.55)	11.0^b^ (0.42)	11.2^b^ (0.31)	11.7^a^ (0.23)	11.6^ab^ (0.49)	11.8^a^ (0.49)	11.6^b^ (0.47)	11.4^b^ (0.51)

*Note*: Mean (standard deviation) followed by the same lowercase letters in the row (same processor) indicates no significant difference at the 5% level (Tukey test or Student's *t*‐test).

**TABLE 3:** Physicochemical and rheological characteristics of wheat flours from different processors (COT, TRA, CIT, NOR, and COU) from the 2022/2023 to 2024/2025 harvests.

Characteristics		Processors of wheat flours/harvests							
		COT^a^	TRA^a^	CIT^b^		NOR^b^			COU^a^
		2022/2023	2023/2024	2022/2023	2023/2024	2022/2023	2023/2024	2024/2025	2023/2024
Alveograph	*P* (mm)	102.1 (16.3)	87.6 (10.8)	89.5^a^ (14.2)	99.8^a^ (18.5)	81.2^b^ (5.5)	88.0^a^ (4.0)	84.2^b^ (3.4)	83.7 (11.6)
	*L* (mm)	69.2 (10.2)	88.1 (12.3)	76.1^b^ (15.4)	92.3^a^ (22.7)	92.2^a^ (24.8)	89.2^a^ (11.4)	91.3^a^ (12.2)	106.8 (15.9)
	*P*/*L*	1.5 (0.45)	1.0 (0.25)	1.3^a^ (0.49)	1.2^a^ (0.48)	0.98^a^ (0.41)	1.0^a^ (0.21)	0.94^a^ (0.18)	0.81 (0.23)
	*W* (× 10^−4^ J)	223.2 (30.9)	205.2 (24.2)	190.5^b^ (27.7)	250.0^a^ (31.1)	189.2^b^ (18.9)	213.3^a^ (24.8)	212.4^a^ (38.0)	248.2 (34.9)
	*Ei* (%)	44.6 (4.1)	40.7 (2.8)	39.5^b^ (4.4)	44.9^a^ (2.6)	40.6^b^ (2.2)	42.4^ab^ (2.5)	44.6^a^ (2.4)	48.8 (4.1)
FN	*FN* (s)	331.8 (31.3)	283.7 (16.8)	292.7^a^ (20.5)	276.8^a^ (28.5)	295.4^a^ (14.3)	294.5^a^ (21.2)	301.9^a^ (11.7)	303.6 (15.8)
Gluten	Wet (%)	31.3 (3.5)	32.8 (0.80)	31.3^b^ (1.50)	33.3^a^ (1.4)	30.8^ab^ (2.7)	28.9^b^ (1.9)	32.3^a^ (2.1)	32.5 (1.5)
	Dry (%)	10.6 (1.2)	10.9 (0.27)	10.2^b^ (0.38)	11.2^a^ (0.46)	10.2^ab^ (1.0)	9.7^b^ (0.63)	11.0^a^ (0.71)	11.1 (0.53)
	Index (%)	96.5 (2.2)	95.1 (1.3)	94.7^b^ (2.2)	97.3^a^ (1.5)	97.6^a^ (0.77)	96.9^ab^ (2.0)	95.4^b^ (1.8)	94.7 (10.5)
Ash	Wet (%)	0.81 (0.20)	0.82 (0.06)	0.84^a^ (0.13)	0.90^a^ (0.11)	0.73^a^ (0.13)	0.69^a^ (0.07)	0.71^a^ (0.07)	0.86 (0.08)
	Dry (%)	0.92 (1.3)	0.94 (0.07)	0.96^a^ (0.14)	1.03^a^ (0.13)	0.84^a^ (0.14)	0.80^a^ (0.08)	0.80^a^ (0.08)	0.99 (0.09)
Color	L*	90.6 (1.3)	89.6 (0.48)	89.7^a^ (0.65)	89.1^a^ (0.62)	91.2^a^ (0.49)	91.4^a^ (0.22)	90.6^a^ (9.1)	90.0 (0.30)
	a*	0.28 (0.04)	0.31 (0.11)	0.18^a^ (0.06)	0.28^a^ (0.02)	−0.11^ab^ (0.17)	−0.09^a^ (0.10)	−0.17^b^ (0.10)	0.18 (0.11)
	b*	10.9 (0.50)	11.3 (0.32)	11.8^a^ (0.39)	11.8^a^ (0.31)	10.4^a^ (0.25)	10.6^a^ (0.28)	10.4^a^ (0.41)	11.8 (0.32)

^a^ Data were not compared statistically because the present results are from only one harvest.

^b^ Mean (standard deviation) followed by the same lowercase letters in the row (same processor) indicates no significant difference at the 5% level (Tukey test or Student's *t*‐test).

The variability observed in dough *P* among processors within the same harvest highlights the influence of raw material and processing conditions on the rheological properties of flour. In 2021/2022, *P* values ranged from 77.31 to 100.45 mm (*p* < 0.05), indicating significant differences in dough resistance to deformation. *P* values for flours from Rio Grande do Sul ranged from 95 to 110 mm, consistent with the mean of 101.6 mm observed in the present study, reinforcing the profile of moderate‐ to high‐strength flours suitable for breadmaking (Wang et al. 2025).

*L*, when analyzed in combination with *P*, provides a better understanding of the flour's technological profile. In 2022/2023, *L* values varied from below 85 mm to above 107 mm among processors, indicating differences in dough extensibility likely related to protein composition and wheat origin. This variability directly affects dough workability during shaping, which is especially important for laminated and molded biscuit production.

The *P/L* ratio proved to be a sensitive indicator of technological balance. Values exceeding 1.2, observed in some processors, indicate more tenacious and less extensible doughs, which may limit spread and expansion in biscuit formulations. Nevertheless, such characteristics can be technologically adjusted through formulation strategies or mixing optimization, underscoring the importance of rheological characterization.

Flour strength (*W*) varied considerably among processors in 2023/2024. Values above 240 × 10^−4^ J suggest greater gluten network stability and gas retention capacity, whereas values below 200 × 10^−4^ J indicate weaker structures that may favor crispness but reduce structural integrity. Therefore, flour selection should be aligned with the specific biscuit type and processing conditions.

The elasticity index (*Ei*) showed correlation with *P* and *L* values. Processors with higher *Ei* exhibited more balanced resistance and extensibility, while lower values suggest less cohesive gluten structures that may require processing adjustments.

Analysis of *FN* values showed inter‐processor differences within the same harvest (*p* < 0.05). In 2023/2024, *FN* ranged from 266 to 307 s, indicating variability in α‐amylase activity. Lower *FN* values may compromise product texture due to excessive starch degradation, whereas higher values reflect reduced enzymatic activity and greater starch integrity.

Wet and dry gluten contents correlated with rheological performance. In 2024/2025, processors with dry gluten values above 11.9% also presented higher *W* and *P*/*L* ratios, indicating stronger gluten networks. Conversely, values below 10.5% were associated with reduced dough strength. The gluten index supported these findings. Although most processors maintained values above 95, one processor showed a significant reduction (87.97) in 2023/2024, suggesting possible protein quality deterioration related to storage or grain condition.

Differences in ash content, particularly in the 2023/2024 harvest, suggest variations in flour extraction rates. Higher ash values indicate greater incorporation of outer kernel layers, while lower values reflect more refined flours suitable for products requiring lighter color and softer texture.

Flour color (L*, a*, and b*) showed discrete yet significant (*p* < 0.05) variation. Higher L* values correspond to lighter flours preferred for uniform biscuit appearance, whereas variations in b* may be associated with carotenoid content and varietal characteristics.

Overall, the integrated analysis demonstrates that, even within the same harvest year, significant technological differences exist among processors. These results reinforce the necessity of processor‐specific quality monitoring and targeted flour selection according to the intended biscuit application. The recent trend toward increased *W* values and stable *FN* suggests improvements in cultivar selection and milling control, contributing to more consistent flour performance for industrial use.

### 3.3 Multivariate Analysis of the Physicochemical and Rheological Profiles of Wheat Flours From Different Processors in Southern Brazil Across the 2021–2025 Harvest Seasons

**FIGURE 5:**
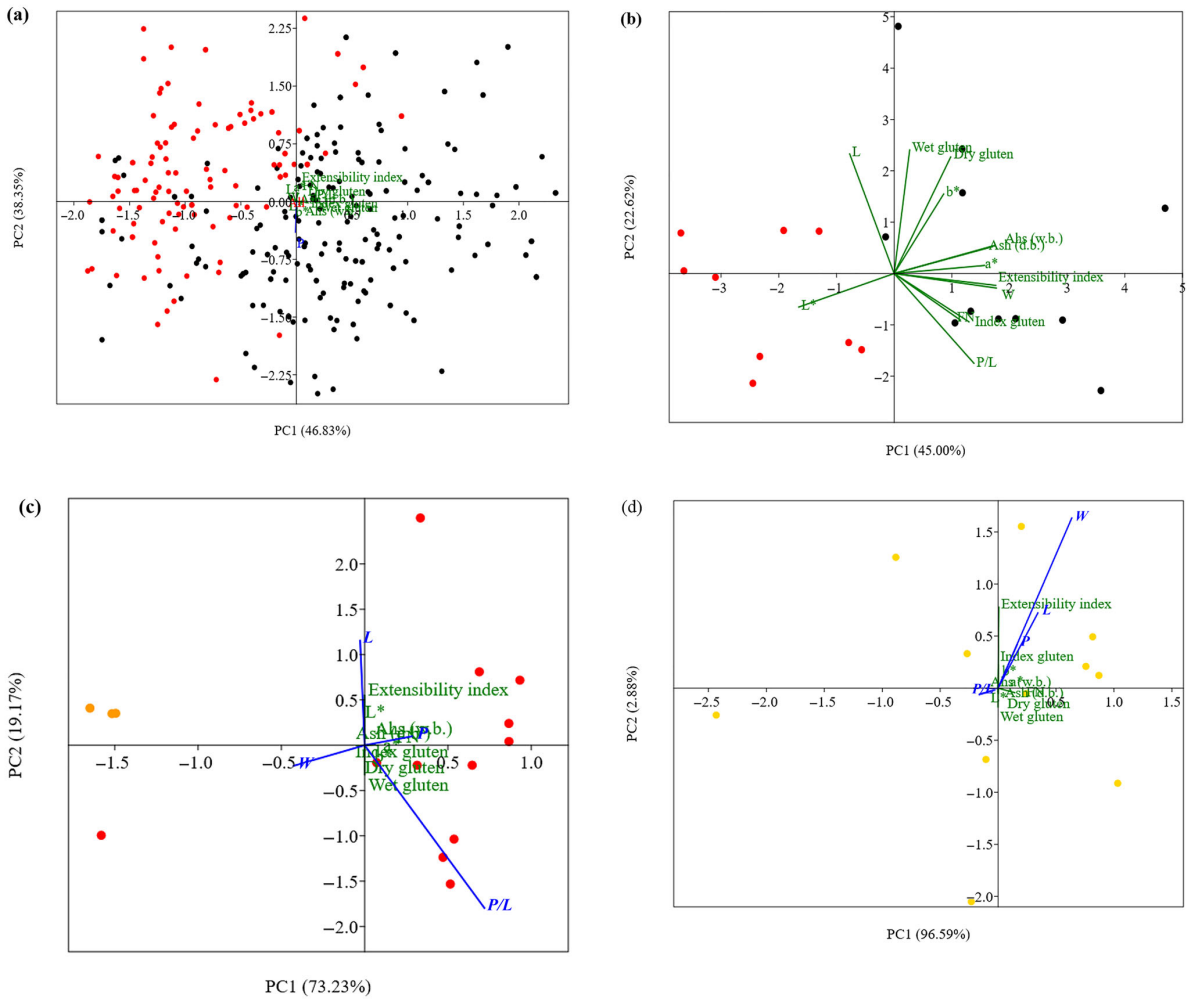
Principal component analysis of the physicochemical and rheological characteristics of wheat flours from processor COO (located in western Paraná State) for the (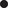) 2021/2022 and (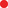) 2022/2023 harvests (a); MOC (located in western Paraná State) for the (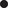) 2021/2022 and (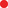) 2022/2023 (b) harvests; COT (located in northwestern Rio Grande do Sul State) (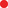) 2022/2023 and (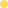) 2023/2024 (c) harvests; and TRA (located in western Paraná State) for the (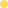) 2023/2024 harvest (d).

**FIGURE 6:**
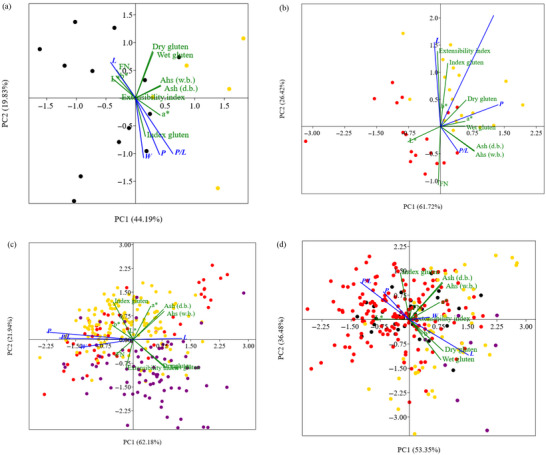
Principal component analysis of the physicochemical and rheological characteristics of wheat flours from processor INF (located in western Paraná State) for the (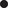) 2021/2022 and (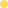) 2023/2024 harvests (a); CIT (located in western Santa Catarina State) for the (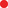) 2022/2023 and (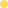) 2023/2024 harvests (b); NOR (located in eastern Paraná State) for the (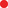) 2022/2023, (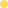) 2023/2024, and (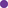) 2024/2025 harvests (c); and ITA (located in western Paraná State) for the (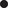) 2021/2022, (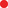) 2022/2023, (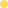) 2023/2024, and (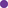) 2024/2025 harvests (d).

**FIGURE 7:**
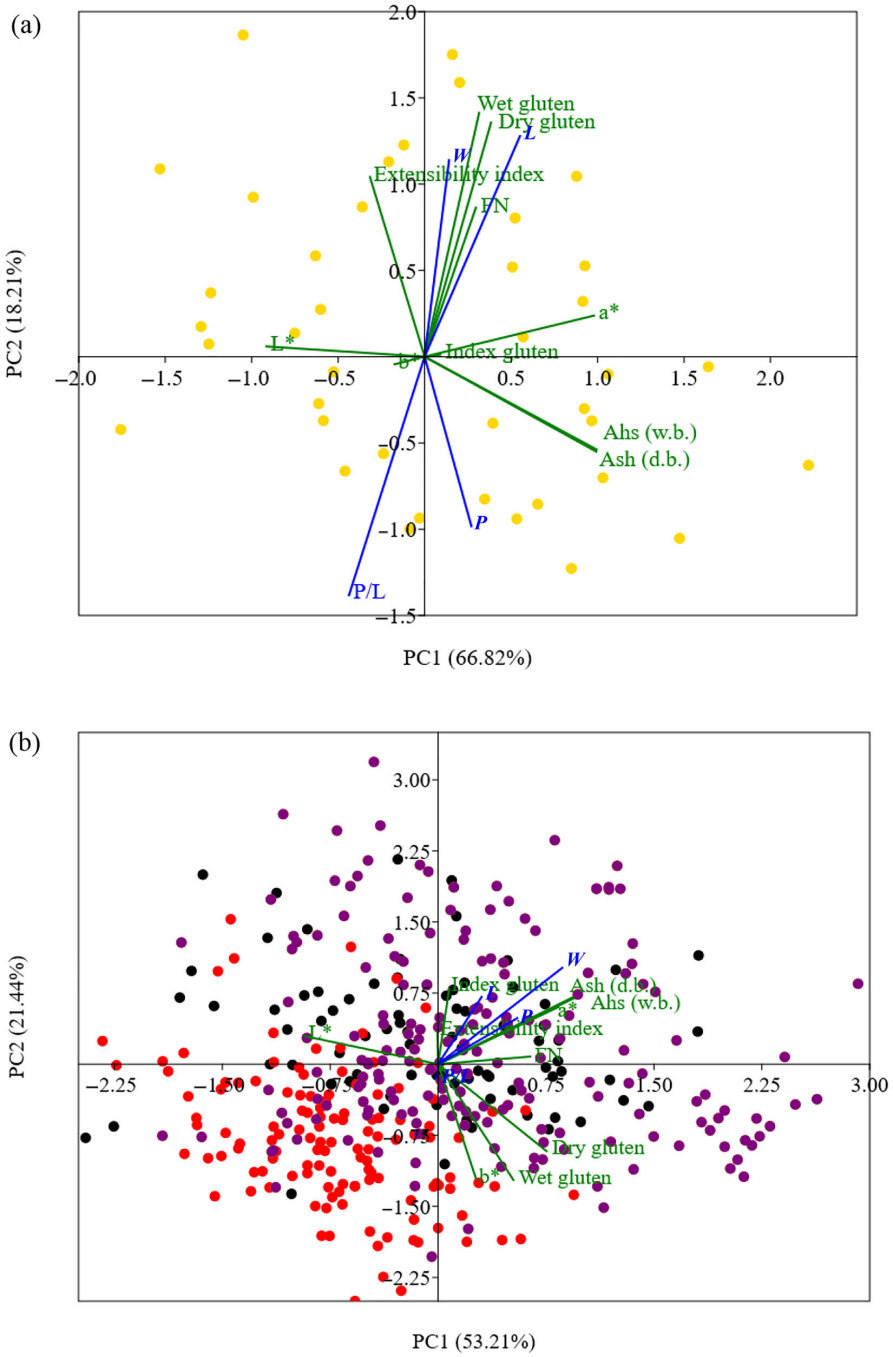
Principal component analysis of the physicochemical and rheological characteristics of wheat flours from processor COU (located in western Paraná State) for the (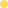) 2023/2024 harvest (a) and from processor TRI (located in central‐western Paraná State) for the (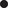) 2021/2022, (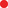) 2022/2023, (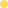) 2023/2024, and (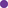) 2024/2025 harvests (b).

PCA (Figures 5‐7) was used to reduce the dimensionality of the physicochemical and rheological data of the flours, highlighting the factors that most contribute to variability among processors and harvests. The analysis showed patterns of clustering and separation based on the characteristics of the flour samples, providing support for understanding the impact of harvest conditions and local industrial practices.

Figure 5a for processor COO (located in the western region of Paraná State) shows marked differences in the rheological parameters (*P*, *L*, and *W*) and color attributes (L* and b*). The 2022/2023 harvest clusters more strongly with high extensibility (*L*) and elasticity index (*Ei*), indicating wheat is more suitable for processes requiring dough conformability. The position of the 2021/2022 harvest toward the vectors associated with dough strength (*W*) and *FN* suggests greater dough resistance and enzymatic stability.

In Figure 5b, referring to processor MOC (Western PR, 2021/2022 vs. 2022/2023), a clear discrimination between harvests is observed. The 2022/2023 harvest exhibited higher L* color values and greater extensibility (*L*), whereas the 2021/2022 harvest presented higher values for the remaining physicochemical and rheological parameters.

For the samples from processor COT, located in northwestern RS, from the 2022/2023 and 2023/2024 harvests, a low level of discrimination was observed. This limited separation may be associated with the reduced number of samples in the 2023/2024 harvest. In contrast, the 2022/2023 harvest displayed a heterogeneous profile with wide data variation (Figure 5c). As only a single harvest (2023/2024) was available for processor TRA (Western PR), the dispersed points in the graph suggest intrinsic variability in both rheological and physicochemical parameters (Figure 5d). This dispersion may be related to batches originating from different wheat sources.

In Figure 6a, for processor INF (Western PR), there is a clear separation between harvests. The 2023/2024 harvest clusters near vectors associated with higher gluten index, ash content, and a* values. The 2021/2022 harvest, on the other hand, displays higher rheological parameters (*W*, *L*, *Ei*, and *FN*) and superior color attributes (L* and b*) of the flour.

In the PCA shown in Figure 6b, clear shifts in the rheological profile are observed between the 2022/2023 and 2023/2024 harvests from processor CIT (located in Western SC). The 2023/2024 harvest stands out for its proximity to the *P/L*, *P*, and *W* vectors, suggesting a dough that is more tenacious and less extensible. Such a profile may reduce dough spread and shaping performance in molded cookies, indicating the need for formulation or process adjustments.

In Figure 6c, a clear temporal trajectory is evident for samples from processor NOR (Eastern PR). The 2024/2025 harvest diverges from the previous ones (2022/2023 and 2023/2024), clustering near vectors associated with wet and dry gluten content and the alveographic index. Meanwhile, the earlier harvests (2022/2023 and 2023/2024) exhibit similar behavior.

For processor ITA (Western PR, 2021/2022 to 2024/2025 harvests), distinct groupings are observed, indicating an evolution in both rheological parameters and color characteristics (Figure 6d). The most recent harvest (2024/2025) is associated with higher extensibility (*L*), increased b* values, and greater wet and dry gluten content, suggesting improved performance in products requiring greater dough elasticity and a more yellowish tone, attributes desirable for certain sensory profiles of biscuits. Conversely, the 2023/2024 harvest, characterized by higher ash content, increased a* values (indicating a redder tone), higher *FN*, and greater gluten strength (*W*), may yield more resistant doughs, potentially affecting final texture and altering product color.

For processor COU (Western PR, 2023/2024), the dispersion of samples around the center of the plot suggests moderate variability within the harvest rather than a strong directional influence of specific variables (Figure 7a). The concentration toward the *P* and *FN* vectors indicates dough with greater resistance and favorable enzymatic stability.

The PCA for processor TRI (Central‐West PR) shows a progressive evolution across harvests, with greater contributions from variables such as *W*, gluten index, and *FN* (Figure 7b). The 2024/2025 harvest stands out with superior performance, suggesting more refined control of milling steps and the selection of wheat with higher baking potential. However, the 2022/2023 and 2023/2024 harvests exhibited similar behavior.

The PCA analysis indicates that, while some industrial units maintain stability in their physicochemical and rheological parameters over the years, others show substantial changes, likely reflecting fluctuations in raw material quality and even improvements in milling processes. More recent harvests, particularly 2024/2025, tended to cluster with desirable attributes such as greater flour strength (*W*), higher gluten index, and enhanced enzymatic stability (*FN*), suggesting progressive technological improvement. These characteristics are especially relevant for the cookie industry, which requires flours with a balanced ratio of tenacity and extensibility, suitable dough conformability, and stability during thermal processing.

Among the processors, ITA and TRI stood out positively, exhibiting a clear trajectory of improvement in the parameters evaluated, with stronger and lighter flours and enhanced rheological performance, particularly in the 2024/2025 harvest. Processors such as COO and INF also showed qualitative improvements over time, associated with increased extensibility and dough stability, attributes beneficial for molded and laminated products. In contrast, processors such as CIT and TRA showed greater intra‐harvest variability, which may require targeted formulation adjustments to ensure final product standardization.

Recent studies have shown that interannual climatic variability (including excessive rainfall, heat anomalies, and humidity fluctuations) can alter starch functionality, α‐amylase activity, and protein–starch interactions, producing measurable shifts in multivariate patterns (Aono et al. 2024; Han et al. 2025). These environmental influences frequently manifest in PCA as harvest‐specific clustering, with wetter or hotter seasons shifting toward vectors associated with changes in *FN*, *P/L* ratio, *W*, or color attributes affected by kernel weathering. Thus, part of the segregation observed among the harvests in this study likely reflects climatic impacts on grain physiology, as documented for southern Brazil in recent years (SECOM 2024), which aligns with global reports linking climate‐driven stress to increased variability in viscoelasticity, enzymatic behavior, and starch integrity.

The PCA also demonstrated that variables such as flour strength (*W*), gluten index, *FN*, and the *P/L* ratio are key determinants in discriminating among processors and harvests, reinforcing the relevance of these metrics as criteria for flour selection in the food industry. The systematic clustering of flours with superior characteristics along these rheological and physicochemical vectors highlights their strategic potential both for supplier selection and qualification and for guiding specific formulations for different biscuit categories, such as fermented, laminated, butter‐type, wafer, and others. This knowledge enables more precise technical adjustments through the addition of improvers, enzymes, and technological aids permitted by current regulations, optimizing industrial performance and the sensory quality of final products.

## 4 Conclusion

Wheat flours commercialized for biscuit production in southern Brazil (2021–2025 harvests) predominantly exhibited technological profiles more suitable for breadmaking. Most samples showed dry gluten >10.5%, *FN* between 266 and 307 s, and *W* frequently >240 × 10^−4^ J, indicating strong gluten networks and medium to high dough strength, conditions that limit extensibility and spread required for biscuit processing.

Significant differences (*p* < 0.05) among processors and harvests were observed mainly for *W*, *P*/*L* ratio, *FN*, and gluten index. PCA confirmed these variables as the primary discriminators of flour quality.

ITA and TRI demonstrated technological improvement in 2024/2025, with better rheological balance and stability, whereas CIT and TRA showed greater intra‐harvest variability. Overall, the results confirm that most flours require adjustment or blending to meet biscuit‐specific requirements, highlighting the importance of monitoring *W*, *FN*, gluten index, and *P*/*L* ratio for supplier selection and industrial standardization.

## Author Contributions

**Andressa Centenaro**: conceptualization, investigation, writing – original draft, methodology. **Eunice Valduga**: data curation, supervision, formal analysis, validation, writing – review and editing. **Clarice Steffens**: data curation, supervision, formal analysis, validation, writing – review and editing.

## Funding

The authors declare no external funding for this study.

## Conflicts of Interest

The authors declare no conflicts of interest.

## Data Availability

The data that support the findings of this study are available from the corresponding author upon reasonable request.
